# Dr. Landon Carter Gray

**Published:** 1900-06

**Authors:** 


Dr. Landon Carter Gray, of New York, died at his home, May 8, 1900, aged 50 years. The *Medical Record* says:

Dr. Gray early turned his attention to the study of diseases of the nervous system, and speedily won for himself an enviable reputation in that line of practice. While in Brooklyn he was professor of neurology at the Long Island College Hospital Medical School, and was visiting neurologist to St. Mary's Hospital. He was one of the founders of the New York Polyclinic, and was the first occupant of the chair of nervous and mental diseases in that institution. He was at various times president of the Medical Society of the County of

New York, the American Neurological Association, the New York Neurological Society and the Society of Medical Jurisprudence, and was for many years chairman of the executive committee of the Congress of American Physicians and Surgeons. He was the author of a work on nervous and mental diseases, and was at one time a frequent contributor to periodical literature in this specialty.

